# Antarctic, Sub-Antarctic and cold temperate echinoid database

**DOI:** 10.3897/zookeys.204.3134

**Published:** 2012-06-25

**Authors:** Benjamin Pierrat, Thomas Saucède, Alain Festeau, Bruno David

**Affiliations:** 1 UMR CNRS 6282 Biogéosciences, Université de Bourgogne, 6 boulevard Gabriel, 21000, Dijon, France

**Keywords:** Southern Ocean, echinoids, Antarctic species, Sub-Antarctic species, cold temperate species

## Abstract

This database includes spatial data of Antarctic, Sub-Antarctic and cold temperate echinoid distribution (Echinodermata: Echinoidea) collected during many oceanographic campaigns led in the Southern Hemisphere from 1872 to 2010. The dataset lists occurrence data of echinoid distribution south of 35°S latitude, together with information on taxonomy (from species to genus level), sampling sources (cruise ID, sampling dates, ship names) and sampling sites (geographic coordinates and depth). Echinoid occurrence data were compiled from the Antarctic Echinoid Database (David et al. 2005a), which integrates records from oceanographic cruises led in the Southern Ocean until 2003. This database has been upgraded to take into account data from oceanographic cruises led after 2003. The dataset now reaches a total of 6160 occurrence data that have been checked for systematics reliability and consistency. It constitutes today the most complete database on Antarctic and Sub-Antarctic echinoids.

## Project details

**Project title:** Macroecology of Southern Ocean echinoids: Distribution, Biogeography and Ecological Niche Modelling.

**Personnel:** Pierrat Benjamin (collection identifier, data collector, data manager, data publisher), Saucede Thomas (collection identifier, data collector), Alain Festeau (computer specialist), David Bruno (collection identifier, general manager).

**Funding.** Phd school E2S Dijon research allowance, CAML/TOTAL, ANR ANTFLOCKS (n°07-BLAN-0213-01), ECOS project (n°C06B02) and BIANZO I and II projects.

**Study area descriptions / descriptor.** The study area covers the Southern Ocean, Sub-Antarctic and cold temperate areas, from the Antarctic continent to 35°S latitude. The aim of the project was to constitute the most complete and consistent echinoid dataset for the Southern Ocean, a vast ocean area that is known for suffering from under-sampling (Griffiths 2010), especially in sectors of East Antarctica, Amundsen and Bellingshausen Seas. The dataset latitudinal range (< 35°S) allows determining possible faunal connections between Antarctic seas and adjacent areas of South America, New Zealand and South Australia.

**Design description:** This dataset was developed to determine the current distribution patterns of Antarctic and Sub-Antarctic echinoid species at the scale of the whole Southern Ocean and to highlight the forcing factors that may control them. The ecological niche modelling (ENM) of 19 echinoid species showed that distribution is mainly structured according to two patterns: (1) a first one represented by species that are not limited to the south of the Polar Front and distributed from the Antarctic coasts to the Sub-Antarctic and cold temperate areas, and (2) a second one with species restricted to the Antarctic area.

In addition, a similarity analysis of echinoid fauna between bioregions of the Southern Ocean was performed at species and genus levels. The analysis reveals faunal connections between southern South America and Sub-Antarctic areas, interpreted as a result of echinoid paleobiogeographic and evolutionary history. Trans-Antarctic faunal connections were also demonstrated and interpreted as a result of West Antarctic Ice Sheet collapses and setting up of trans-Antarctic seaways during the Pleistocene.

Among the environmental parameters that may control echinoid distribution, three parameters seem to be the main forcing factors of echinoid distribution: depth, sea-ice cover and sea surface temperature. However, the respective contributions of these parameters vary among species. Differences are particularly emphasized in the case study of the genus *Sterechinus*, *Sterechinus neumayeri* being the species the most dependent on environmental conditions that prevail along the Antarctic coasts (sea surface temperature and sea-ice cover), while *Sterechinus antarcticus* does not seem to be so much under the control of these parameters. However, *Sterechinus antarcticus* is not present over the whole area of its potential distribution, what can be explained as the result of either (1) oceanographic factors (role of the Polar Front as a biogeographic barrier), (2) biotic interactions (inter-specific competition) or (3) temporal contingencies (ongoing range expansion).

## Taxonomic coverage

**General taxonomic coverage description:** This database is devoted to all echinoid species inhabiting ocean areas south of 35S latitude (Echinodermata: Echinoidea). Echinoids are well represented in the Antarctic benthic communities in terms of frequency, abundance and species richness. They are frequently collected both at shallow depths over the continental shelf and in deeper waters of the continental slope and ocean basins. With 82 species ever described that represent about 10% of echinoid species worldwide, the Southern Ocean is particularly rich in echinoid species. The Antarctic echinoid fauna is characterised by a relative high morphological diversity and high rate of endemism (66% of species - David et al. 2005b). It should be noticed that Antarctic echinoid diversity is represented by a few orders (7) among which the two orders Spatangoida and Cidaroida include 64.6% of Antarctic species. As a comparison, South Australian and New Zealand areas comprise 113 echinoid species, 62 genera and 12 orders, while southern South America only 36 species, 23 genera and 8 orders, and the Southern Ocean 82 species, 30 genera and 7 orders. Identifications and taxonomic accuracies were based on Mortensen (1928, 1935, 1943, 1950, 1951) and David et al. (2005b).

### Taxonomic ranks

**Kingdom**: Animalia

**Phylum**: Echinodermata

**Class**: Echinoidea

**Order**: Arbacioida, Cassiduloida, Cidaroida, Clypeasteroida, Echinoida, Echinothurioida, Holasteroida, Pedinoida, Salenoida, Spatangoida, Temnopleuroida.

**Family**: Apatopygidae, Arachnoididae, Arbaciidae, Aspidodiadematidae, Asterostomatidae, Brissidae, Cidaridae, Clypeasteridae, Diadematidae, Echinidae, Echinolampadidae, Echinometridae, Echinothuriidae, Fibulariidae, Hemiasteridae, Laganidae, Loveniidae, Mellitidae, Palaeotropidae, Pedinidae, Phormosomatidae, Plexechinidae, Pourtalesiidae, Saleniidae, Schizasteridae, Spatangidae, Temnopleuridae, Toxopneustidae, Urechinidae.

## Spatial coverage

**General spatial coverage:** The sampling area ranges from 35°S to 71°S latitude and from 180°W to 180°E longitude. The 35°S limit is coincident with the position of the Subtropical Convergence (Tchernia 1980; Knox 1983), which is considered to determine the limit between tropical and cold temperate marine species. The latter species were considered in the database, as they are likely to interact with Antarctic species in the future according to scenarii of forthcoming global climate change or to have interacted with them in the past.

**Coordinates:** 71°0'0"S and 35°0'0"S Latitude; 180°0'0"W and 180°0'0"E Longitude.

## Temporal coverage

1872–2010.

## Natural collections description

**Parent collection identifier:** Pierrat Benjamin, David Bruno, Saucede Thomas

**Collection name:** Antarctic, Sub-Antarctic and cold temperate echinoid database

**Collection identifier:** Pierrat Benjamin, David Bruno, Saucede Thomas

**Specimen preservation method:** Alcohol

## Methods

**Method step description:** see sampling description above.

**Study extent description:** The study area includes the Antarctic, Sub-Antarctic and cold temperate regions. Five regions are particularly focussed on: (1) the Southern Ocean with the Antarctic Peninsula, the South Orkney Island, the Weddell Sea, Dronning Maud Land, Enderby Land, the Mawson Sea, Adelie Land, the Ross Sea, the Amundsen Sea and the Bellingshausen Sea, (2) the Sub-Antarctic Islands composed of Prince Edward, Crozet, Bouvet, Kerguelen and Heard Islands, (3) the South American coast, with the Argentinean coast, the Clilean coast and the Falkand Island, (4) the New Zealand coast and (5) the South Australian coast inclusive of Tasman coast.

**Sampling description:** Echinoids were collected during oceanographic cruises led in the Southern Ocean from 1872 to 2003. The database has been upgraded with data collected from 2003 to 2010. Sample depth ranges go from the shoreline to the deep sea. Sampling was performed with different protocols and different gears, specific to each cruise (Agassiz Trawl, Box Core, Beam Trawl, Epibenthic Sledge…). Each echinoid sample was separated at sea from other specimens of the macrofauna, then identified and fixed in formaldehyde for old samples, in 100% ethanol for recent ones.

**Quality control description**: Systematics reliability and consistency have been checked for by Bruno David, Thomas Saucède and Benjamin Pierrat, identification being based on species descriptions produced by Mortensen (1928, 1935, 1943, 1950, 1951) for Australian, New Zealand and South American species, on *Synopses of the Antarctic benthos* by David et al. (2005a) for Antarctic species.

**Data resources**: The data underpinning analyses of the paper are deposited at GBIF, the Global Biodiversity Information Facility, http://ipt.biodiversity.aq/archive.do?r=antarctic_Sub-Antarctic_and_cold _temperate_echinoid_database

## Datasets

**Dataset description**: Our knowledge on Antarctic echinoids have been synthesized by David et al. (2005a) and led to constituting a first database: *Antarctic echinoids: an interactive database* (David et al. 2005b). This database listed echinoid samples collected during Antarctic cruises led before 2003 and counted 2029 occurrence records that ranged from 70°S to 45°S latitude. Research data to upgrade this first database focused on different regions: (1) The Southern Ocean with samples collected during Antarctic cruises led after 2003, allowing the densification of data for under-sampled areas such as the eastern coast of Antarctica, the Bellingshausen and the Amundsen Seas (Griffiths 2010); (2) South America, with samples from Argentinian cruises housed at the Museo Argentino de Ciencas Naturales (Buenos Aires-ARG); (3) New Zealand and South Australia with samples from different collections (Australian Museum, Sydney, AUS; Melbourne Museum, Melbourne, AUS and NIWA, Wellington, NZ). Systematics has been checked for following Mortensen (1928,^1935^, ^1943^, ^1950^, ^1951^) for Australian, New Zealand and South American species, and the *Synopses of the Antarctic benthos* by David et al. (2005a) for Antarctic species. All data are georeferenced (WGS1984).

**Object name:** Darwin Core Archive Antarctic, Sub-Antarctic and cold temperate echinoid database

**Character encoding:** UTF-8

**Format name:** Darwin Core Archive format

**Format version:** 1.0

**Distribution:** http://ipt.biodiversity.aq/archive.do?r=antarctic_SubAntarctic_and_cold_temperate_echinoid_database

**Publication date of data:** 2012-03-26

**Language:** English

**Metadata language:** English

**Date of metadata creation:** 2012-03-26

**Hierarchy level:** Dataset
